# Structural and quantitative evidence of α2–6-sialylated *N*-glycans as markers of the differentiation potential of human mesenchymal stem cells

**DOI:** 10.1007/s10719-016-9699-6

**Published:** 2016-06-17

**Authors:** Kayo Hasehira, Jun Hirabayashi, Hiroaki Tateno

**Affiliations:** 1 Biotechnology Research Institute for Drug Discovery (BRD), Tsukuba Central 2, 1-1-1 Umezono, Tsukuba, Ibaraki, 305-8568 Japan; 2 National Institute of Advanced Industrial Science and Technology (AIST), Tsukuba Central 2, 1-1-1 Umezono, Tsukuba, Ibaraki, 305-8568 Japan

**Keywords:** Mesenchymal stem cell, Glycan, Differentiation, Sialylation, Hydrazinolysis

## Abstract

Human somatic stem cells such as mesenchymal stem cells (hMSCs) have the capacity to differentiate into mesenchymal tissue lineages and to alter immune regulatory functions. As such, they hold promise for use in stem cell-based therapies. However, no method is currently available to evaluate the actual differentiation capacity of hMSCs prior to cell transplantation. Previously, we performed a comprehensive glycan profiling of adipose-derived hMSCs using high-density lectin microarray and demonstrated that *α*2–6-sialylation is a marker of the differentiation potential of these cells. Nevertheless, no information was available about the structural details of these of *α*2–6-sialylated glycans. Here we used high performance liquid chromatography (HPLC) analysis combined with mass spectrometry (MS) to perform a structural and quantitative glycome analysis targeting both *N*- and *O*-glycans derived from early (with differentiation ability) and late (without differentiation ability) passages of adipose tissue-derived hMSCs. Findings in these cells were compared with those from human induced pluripotent stem cells (hiPSCs), human dermal fibroblasts (hFibs) and cartilage tissue-derived chondrocytes. A higher percentage of *α*2–6-sialylated *N*-glycans was detected in early passage cells (24–28 % of sialylated *N*-glycans) compared with late passage cells (13–15 %). A major *α*2–6-sialylated *N*-glycan structure detected in adipose-derived hMSCs was that of mono-sialylated biantennary *N*-glycan. Similar results were obtained for the cartilage tissue-derived chondrocytes, Yub621c (28 % for passage 7 and 5 % for passage 28). In contrast, no significant differences were observed between early and late passage hMSCs with respect to *α*2–6-sialylated *O*-glycan percentages. These results demonstrate that levels of *α*2–6-sialylated *N*-glycans, but not *O*-glycans, could be used as markers of the differential potential of hMSCs.

## Introduction

Human mesenchymal stem cells (hMSCs) isolated from tissues such as adipocyte, bone marrow, and umbilical cord blood [1, 2] are attractive cell therapy products (CTPs) for use in regenerative medicine based on their abilities to self-renew and to differentiate into mesenchymal tissue lineages such as osteoblasts, cartilage, and adipocytes [3, 4]. This is in addition to their capacity to alter immune regulatory functions [5]. Indeed, hMSCs have already been applied as CTPs to treat patients with diseases such as graft-*versus*-host disease (GVHD), acute cardiac insufficiency, critical limb ischemia, osteoarthritis, spinal injury, and diabetes [6, 7]. hMSCs are obtained from the bone marrow or other tissues of donors from different backgrounds in terms of age, sex, race etc. These cells are then expanded *in vitro* until a required cell number is reached, and are then transplanted into patients following the necessary cell treatment protocols. A complicating factor is that hMSCs are heterogeneous cell populations, whose properties vary depending on the tissues and donors from which they are derived, isolation methods, culture conditions, and culture passages. Therefore, the development of quality control systems with respect to hMSC handling and use is critical to ensure that the appropriate therapeutic effects as well as the safety of CTPs is achieved. To this end, no cell surface markers are currently available to evaluate the differentiation potential of stem cells, this being one of the most important measures of the possible therapeutic effects of hMSCs.

To identify cell surface glycan markers that might enable the differentiation potential of hMSCs to be assessed, we build here on glycome analysis work previously carried out on different passages of adipose-derived hMSCs using high-density lectin microarray [8]. We found that *α*2–6-linked sialic acid (*α*2–6Sia)-specific lectins showed stronger binding to early passage adipose-derived hMSCs with differentiation ability to adipocytes and osteoblasts than did late passage cells without the ability. Flow cytometry analysis also supported the results obtained by lectin microarray. While similar results were obtained for bone marrow-derived hMSCs and cartilage tissue-derived chondrocytes, in the case of human dermal fibroblasts (hFibs), however, which did not have differentiation potential, but did possess a high proliferation ability, no binding of *α*2–6Sia-specific lectins was observed. This finding demonstrates that the binding of *α*2–6Sia-specific lectins is associated with the differentiation ability of cells, but not to their capacity to proliferate. While this may be the case, no information is currently available concerning the detailed glycan structures of early passage hMSCs (with differentiation ability) compared to late passage cells (without the ability to differentiate).

In this study, we provide structural data for *N*- and *O*-glycans derived from early and late passages of human adipose tissue-derived hMSCs and cartilage tissue-derived chondrocytes. For quantitative comparison, glycans were liberated by gas-phase hydrazinolysis from cells fluorescently tagged with 2-aminopyridine at their reducing terminus, following which of the derived pyridylaminated (PA-) glycans were purified by multiple-mode high-performance liquid chromatography (HPLC). The structures of the PA-glycans were determined and quantified by HPLC mapping in conjunction with matrix-assisted laser desorption-ionization time-of-flight mass spectrometry (MALDI-TOF MS) and exoglycosidase digestion analyses. We demonstrate that a higher percentage of *α*2–6-sialylated *N*-glycans is expressed in early passage hMSCs compared with late passage cells, whereas no significant difference in the percentage of *α*2–6-sialylated *O*-glycans was observed between early and late passage hMSCs. These results demonstrate that *α*2–6-sialylated *N*-glycans could serve as markers of the differential potential of hMSCs.

## Materials and methods

### Cells

hMSCs derived from adipose tissues (Life technologies, lot#: 2117, 2118), cartilage tissue-derived chondrocytes from polydactylous human fingers (Yub621c, RIKEN CELL BANK, #: RBRC-HMS0013), and hFibs (ATCC, #: PCS-201-012, Lot: 58,605,481) were cultured in 10 mL of MesenPRO RS™ Medium (GIBCO, Cat#: 12,746–012) supplemented with 2 mM L-glutamine and 1 % penicillin–streptomycin on 10 cm cell culture dishes. Cells were seeded at 3 x 10^5^ cells per dish and sub-cultured at 80 % confluency as per the manufacturer’s instructions. 201B7 hiPSCs were cultured in 2.5 mL of mTeSR1 (STEMCELL Technologies) on 6-well plates. Cells were counted with a TC20 Automated Cell Counter (Bio-Rad).

### Materials

Acetonitrile and 1-butanol were obtained from Nacalai Tesque. Acetic acid was purchased from Wako. Dowex 50WX2 (200–400 mesh, H^+^ form) was obtained from Muromachi Technos Co., Ltd. GlycoTAG reagent kit was from Takara Bio. 2, 5-dihydroxybenzoic acid was from Bruker Daltonics. The Sep.-PAK Plus C18 cartridge was from Waters. The Mono Q 5/5 HR column (5.0 x 50 mm), the PALPAK Type-R column (4.6 x 250 mm) and the Shodex Asahipak NH2P-50 4D column (4.6 x 150 mm) were from GE Healthcare Bio-Sciences Corp, Takara Bio, and Showa Denko, respectively. *α*2–3, –6- sialidase (*Clostridium perfringens*) was from Merck, and *α*2–3-sialidase cloned from *Salmonella typhimurimum* LT2 and expressed in *Escherichia coli* was from Takara Bio. *β*-galactosidase, *β*-*N*-acetylhexosaminidase, and *α*-L-fucosidase were from ProZyme, Inc. Standard glycans such as core-fucosylated biantennary *N*-glycan (Cat#: 4109), asialo (Cat#: 4101), mono- (Cat#: 4122), di- (Cat#: 4123), tri- (Cat#: 4124), and tetra-sialylated *N*-glycans (Cat#: 4125). were obtained from Takara.

### Release of *N*- and *O*-glycans and pyridylamination

Protein glycans were released from cells by gas-phase hydrazinolysis [9–11] using Hydraclub Y2100 (J-Oil Mills, Inc., Tokyo, Japan). Cells (approximately 1 x 10^6^ cells) were extensively lyophilized in vacuo, and treated with anhydrous hydrazine at 100 °C for 4 h. After the reaction, the anhydrous hydrazine was removed in vacuo. The released glycans were re-*N*-acetylated with addition of acetic anhydride in a saturated sodium bicarbonate solution, desalted with Dowex 50WX2, and lyophilized [9]. The reducing ends of the liberated glycans were tagged with 2-aminopyridine using GlycoTAG (Takara Bio) and the GlycoTAG reagent kit [12–14]. The bulk of excess reagents in the reaction mixture was removed by phenol/chloroform extraction [15] and subsequent solid-phase extraction using a Sep-PAK Plus C18 cartridge [16]. The PA-glycans were analyzed by HPLC.

### HPLC

Anion-exchange HPLC was performed on a Mono-Q column at a flow rate of 1.0 ml/min using two eluents, A and B. Eluent A was distilled water titrated to pH 9.0 with 1 M aqueous ammonia, and eluent B was a 0.5 M ammonia acetate solution titrated to pH 9.0. The column was equilibrated with 100 % eluent A. After injecting a sample, the proportion (*v*/v) of eluent B was increased linearly to 10 % (3 min), then to 40 % (14 min), and finally to 100 % (5 min). The fluorescence of the eluted PA-glycans was detected with excitation and emission wavelengths of 310 nm and 380 nm, respectively.

Size-fractionation HPLC was performed on a Shodex Asahipak NH2P-50 4D column (4.6 x 150 mm) at a flow rate of 1.0 ml/min at 25 °C with a gradient system: two eluents, C and D, were used, where eluent C was acetonitrile: water: acetic acid (970:70:3, *v*/v/v) titrated to pH 7.0 with 7 M aqueous ammonia, and eluent D was acetonitrile:water:acetic acid (200:800:3, *v*/v/v) titrated to pH 7.0 with 7 M aqueous ammonia. The column was equilibrated with 95 % eluent C and 5 % eluent D. After injection of a sample solution, the proportion (*v*/v) of eluent D was increased linearly from 5 % to 33 % (3 min), then to 100 % (75 min). Glycan fluorescence was detected at excitation and emission wavelengths of 320 nm and 400 nm, respectively.

Reversed-phase HPLC was performed on a PALPAK Type-R column (4.6 x 250 mm) with two eluents, E and F, where eluent E was 100 mM ammonium acetate, pH 4.0, and eluent F was 100 mM ammonium acetate, pH 4.0, containing 0.5 % (*v*/v) 1-butanol. The column was equilibrated with 75 % eluent E and 25 % eluent F. After injection of a sample, the proportion (*v*/v) of eluent F was increased linearly from 25 % to 100 % (60 min) at a flow rate of 1.0 ml/min at 25 °C. Glycan fluorescence was detected at excitation and emission wavelengths of 320 nm and 400 nm, respectively.

### MS

The purified PA-glycans were subjected to analysis by MALDI-TOF MS with an Ultraflex mass spectrometer (Bruker Daltonics, Bremen, Germany), equipped with a 337-nm nitrogen laser, and set at 20 kV extraction voltage. 2, 5-dihydroxybenzoic acid (1 mg/ml in 30 % ethanol) was used as the matrix. Analyses were carried out in reflector mode over a mass range of *m*/*z* 0 to 2000, or 1000 to 4000 in the positive ion mode. Each spectrum was measured by 150 laser shots.

### Quantification of PA-saccharides

Each PA-glycan was quantified by the peak area compared with that corresponding to an appropriate authentic standard separated under the same HPLC conditions. PA-GlcNAc was used as the authentic calibration standard. Relative yields were expressed as percentages compared to the total amounts of *N*-linked or *O*-linked glycans prepared from each of the cell samples.

### Exoglycosidase treatment

Exoglycosidase treatment of PA-glycans was carried out by using *α*2–3, –6-sialidase (*C. perfringens*: Merck), *α*2–3-sialidase (*S. typhimurimum* LT2, recombinant, *E. coli*: Takara Bio), *β*-galactosidase (Jack bean: ProZyme, Inc.), *β*-*N*-acetylhexosaminidase (Jack bean: ProZyme, Inc.), or *α*-L-fucosidase (bovine kidney: ProZyme, Inc.). PA-glycan was treated with the enzyme (50 mU) in 20 μl of 50 mM ammonium acetate buffer (*β*-galactosidase: pH 3.5, *α*2–3, −6-sialidase and *β*-*N*-acetylhexosaminidase: pH 5.0, *α*2–3-sialidase and *α*-L-fucosidase: pH 5.5), at 37 °C for appropriate periods (0.5 h for *α*2–3, –6-sialidase and *α*2–3-sialidase; 20 h for others). The enzyme reaction was terminated by heating at 99.9 °C for 5 min.

### Analysis of the Sia linkage type

The sialic acid linkage type was determined by its sensitivity to *α*2–3, −6-sialidase (*C. perfringens*, Merck) and *α*2–3-sialidase (*S. typhimurimum* LT2, recombinant, *E. coli*, Takara Bio) treatment. Each acidic saccharide was treated with both sialidases, and the products were analyzed by anion-exchange HPLC using a Mono-Q column. If an acidic glycan has the *α*2–3 type of sialic acid linkage, its retention time should decrease after digestion both with *α*2–3, −6 and *α*2–3-sialidases. In contrast, if the glycan has the *α*2–6 type linkage, its retention time should be preserved after *α*2–3-sialidase treatment. When all Sia residues are removed by the action of *α*2–3, −6-sialidase, a neutral glycan should be generated.

## Results

### Basic strategy of glycan analysis of adipose-derived hMSCs

*N*- and *O*-glycans were liberated by gas-phase hydrazinolysis from early and late passages of lyophilized adipose-derived hMSCs. Two lots of early and late passages of adipose-derived hMSCs (lot#: 2117, passage 5 (P5) and P26; lot#: 2118, P3 and P28) were analyzed to confirm the reproducibility and consistency of the results. Whole glycans comprising both intracellular (ER and Golgi) and surface membranous fractions were re-*N*-acetylated and labeled with 2-aminopyridine.

**Fig. 1:**
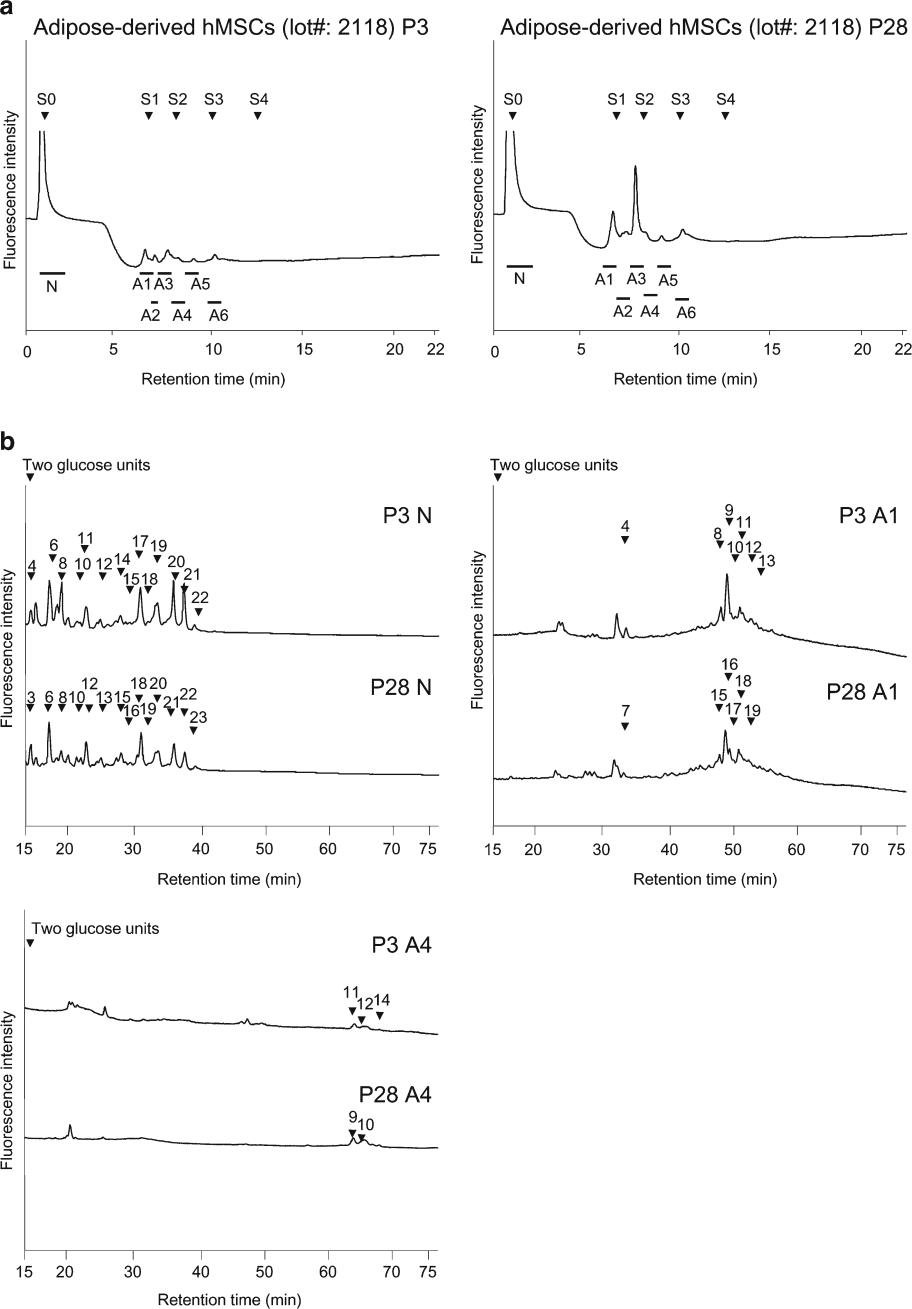
Comparison of HPLC profiles of glycans derived from adipose-derived hMSCs lot#: 2118 P3 and P28. **a** Anion-exchange HPLC profiles. S0, S1, S2, and S3 are standard *N*-glycans containing 0, 1, 2, and 3 Sia residues. **b** Size-fractionation HPLC profiles of neutral fraction (N), monosialylated *N*-glycan fraction (A1), and disialylated *N*-glycan fraction (A4). Following anion-exchange HPLC, further purification was performed. Each peak was pooled, and when necessary, was further purified by reversed-phase HPLC, or subjected to further structural analysis. Arrowheads indicate the peak numbers

The derived PA-glycans were first separated by anion-exchange HPLC (Fig. 1a). Neutral glycans, which passed through the column, were pooled and designated the “N″ fraction. Adsorbed anionic glycans were fractionated and designated the “A1-A6” fractions (according to the order of elution, Fig. 1a). These fractions were then subjected to size-fractionation HPLC (for fractions N, A1 and A4, see Fig. 1b). Each peak was fractioned, and when necessary, the eluate was further purified by reversed-phase HPLC (chromatogram not shown). Each purified PA-glycan was quantified on the basis of fluorescence intensity relative to an appropriate authentic standard (PA-GlcNAc).

The purified neutral glycans (No.1–16 and 28–29) were analyzed by MALDI-TOF MS and reversed-phase HPLC (chromatogram not shown), and when necessary, were treated with glycosidases to confirm their structure. As a result, unambiguous assignment was made for all glycans, with 16 *N*-glycans and 2 *O*-glycans identified as neutral glycans in four different cell lines (Tables 1 and 2).

**Table 1:**
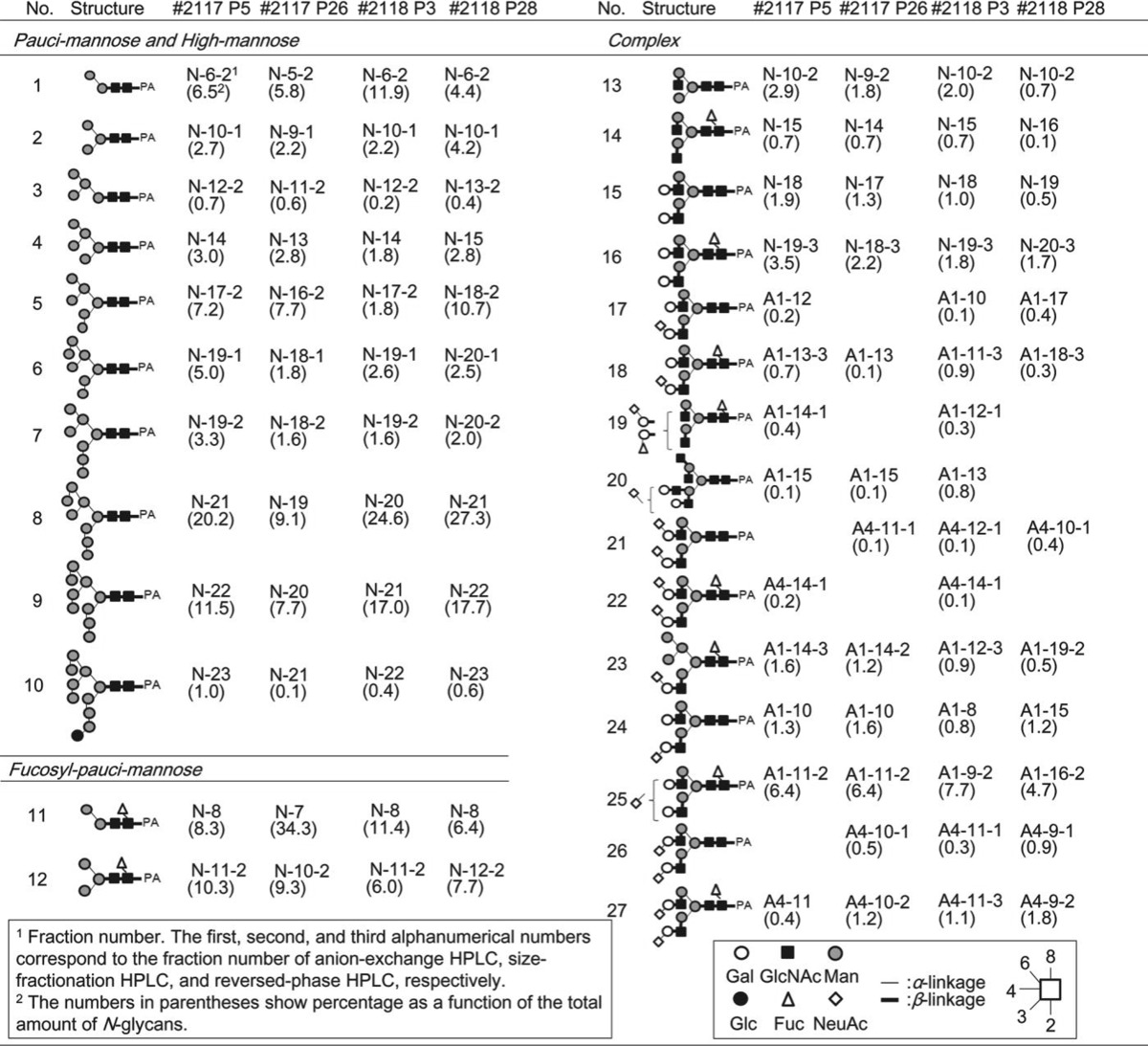
List of *N*-glycans identified in adipose-derived hMSCs

**Table 2:**
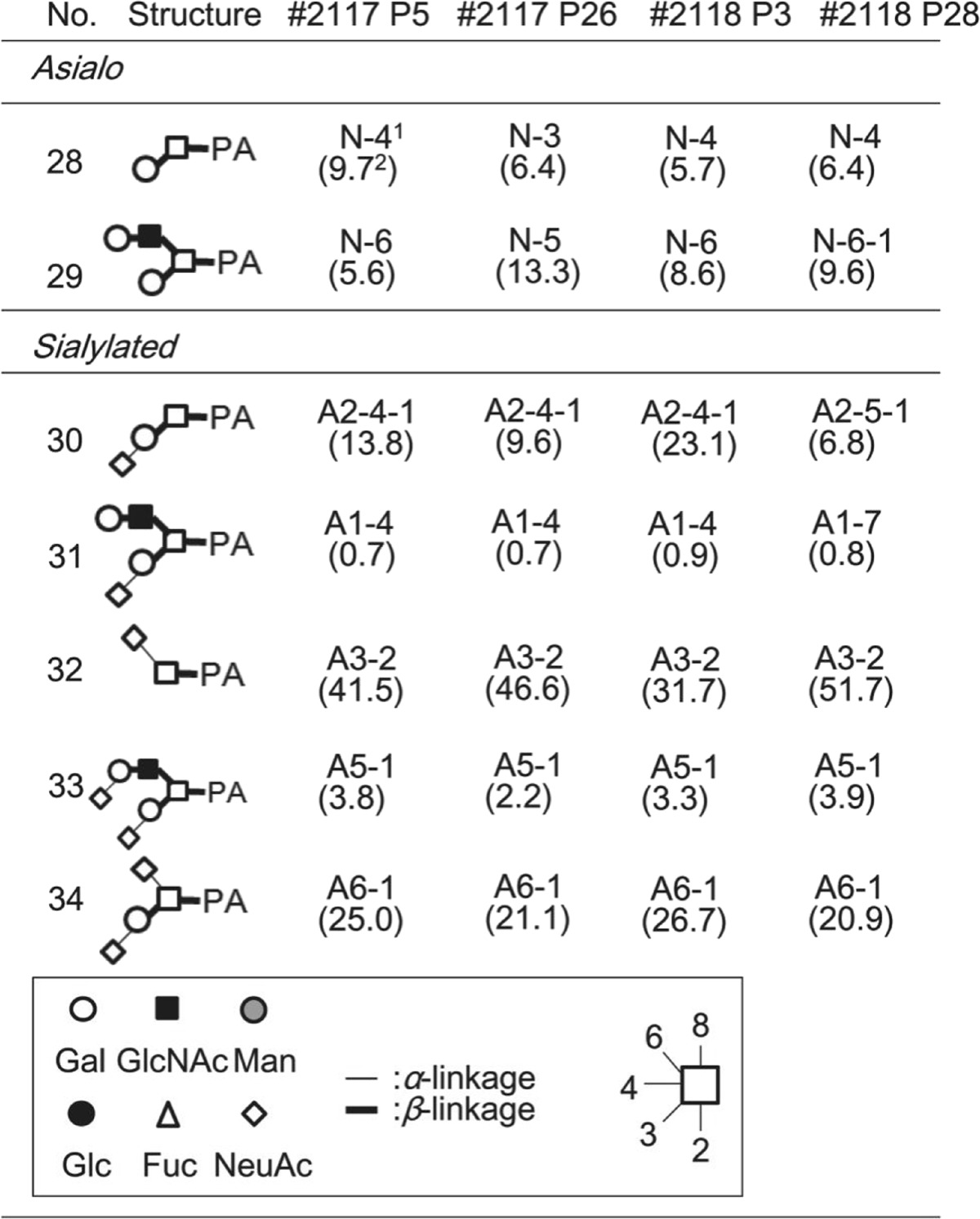
List of *O*-glycans identified in adipose-derived hMSCs

^1^Fraction number. The first, second, and third alphanumerical numbers correspond to the fraction number of anion-exchange HPLC, size-fractionation HPLC, and reversed-phase HPLC, respectively.

The numbers in parenthesis show percentage as a function of the total amount of *O*-glycans.

Acidic glycans (A1-A6 fractions) were analyzed by a combination of sialidase digestion, MALDI-TOF MS and reversed-phase HPLC. After digestion with universal α2–3, −6-sialidase, the products were passed through a Mono-Q column, analyzed by MALDI-TOF MS and reversed-phase HPLC. Their structures were determined as described above. The type of linkage of each sialic acid was determined by susceptibility to two sialidases, *i.e*., α2–3-specific sialidase and the universal α2–3, −6-sialidase. For example, the structure of α2–6-disialylated, core-fucosylated biantennary *N*-glycan, A4–14-1, derived from adipose-derived hMSCs (Lot#:2117 P5) was determined as follows. When A4–14-1 was treated with α2–3, −6-sialidase (*C. perfringens*, Merck) and analyzed by MALDI-TOF MS, the glycan was determined as the monofucosylated biantennary glycan (*m*/*z*, 1865.5 [M + H]^+^ in positive mode), which exactly matched with the elution time of the standard core-fucosylated biantennary *N*-glycan by reversed-phase HPLC. On the other hand, when A4–14-1 was treated with α2–3-sialidase, no Sia was released, demonstrating that the linkage mode of Sia of A4–14-1 is α2–6Sia. Disialylation on both branches of biantennary *N*-glycans could be confirmed by comparing with the retention time with standard glycans of asialo, mono-, di-, tri-, and tetra-sialylated *N*-glycans in an anion-exchange chromatography.

As a result, 9 and 8 acidic *N*-glycans were identified from P5 and P26 of adipose-derived hMSCs (lot#: 2117), respectively, while 11 and 8 acidic *N*-glycans were identified from lot#: 2118 P3 and P28, respectively (Table 1). Five acidic *O*-glycans were identified in A1–6 fractions obtained from four different cell lines (Table 2). Finally, 25, 24, 27, and 24 *N*-glycans were identified from P5 and P26 of adipose-derived hMSCs from lot#: 2117, and P3 and P28 of adipose-derived hMSCs from lot#: 2118, respectively (Table 1). Seven *O*-glycan structures were identified from the cell lines (Table 2).

### Comparative analysis of *N*- and *O*-glycans between early and late passages of adipose-derived hMSCs

**Fig. 2:**
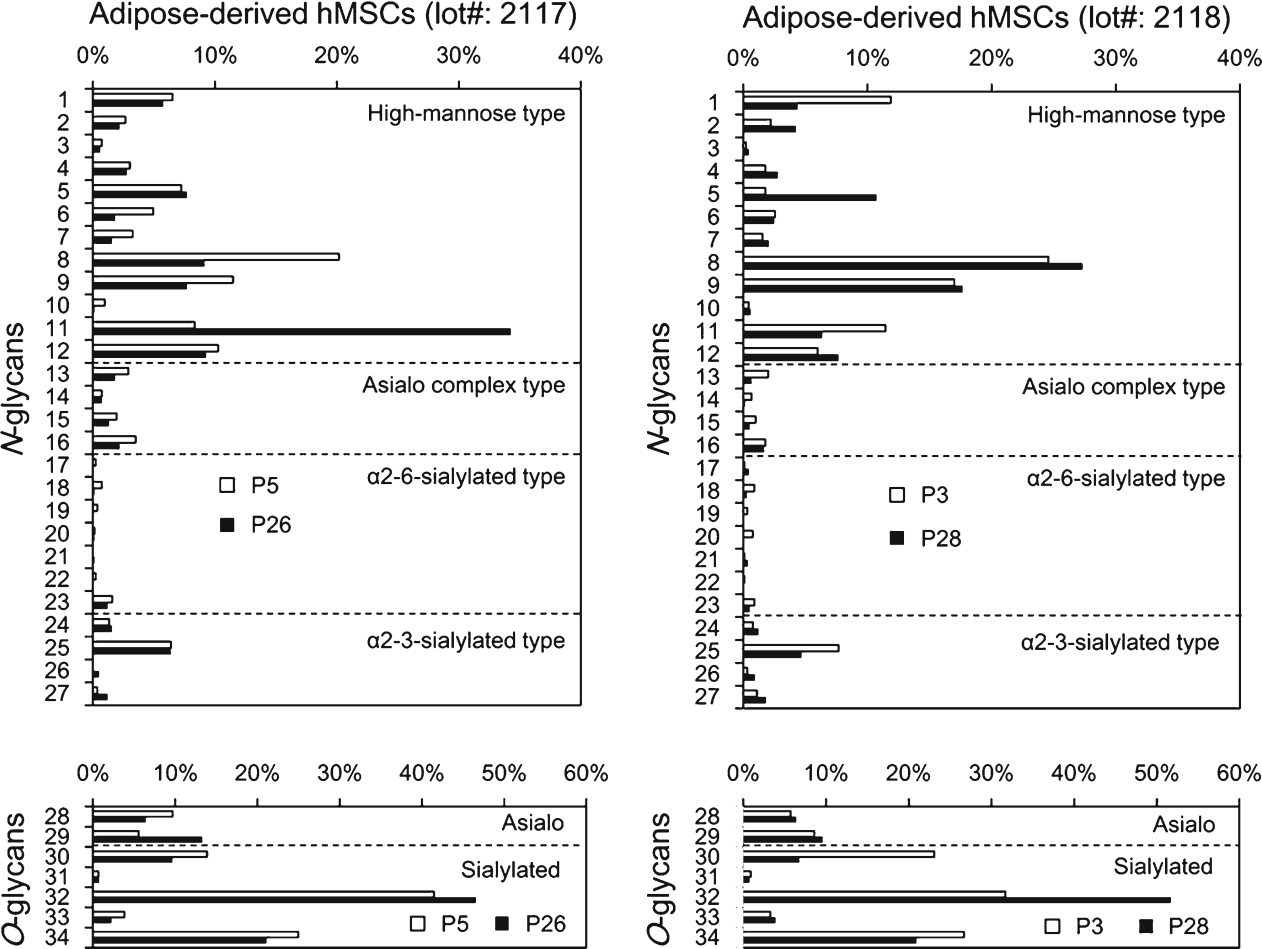
Comparison of *N*- and *O*-glycans prepared from adipose-derived hMSCs lot#: 2117 and lot#: 2118. Each glycan is expressed as a percentage of the total amount of *N*- or *O*-glycans obtained from each sample (taken as 100 %). Glycan numbers correspond to those used in Tables 1 and 2

We next compared the percentage of each glycan derived from early and late passages of adipose-derived hMSCs, taking the total amounts of *N*- or *O*-glycans prepared from each sample to be 100 % (Fig. 2). Among *N*-glycans, high-mannose type *N*-glycans (No.1–12) accounted for a substantial fraction in both the early and late passages of these cells. Interestingly, the expression patterns of high-mannose type *N*-glycans were significantly different between the two lots. In terms of No. 8 glycan, for example, early passage cells (P5) of adipose-derived hMSCs (lot#: 2117) exhibited higher percentage than late passage cells (P26), whereas late passage cells (P28) gave higher percentage than early passage cells (P3) in lot#: 2118. In terms of No. 11 glycan, late passage cells (P26) exhibited much higher percentage than early passage cells (P5) of adipose-derived hMSCs (lot#: 2117), whereas early passage cells (P3) gave higher percentage than late passage cells (P28) in lot#: 2118.

**Fig. 3:**
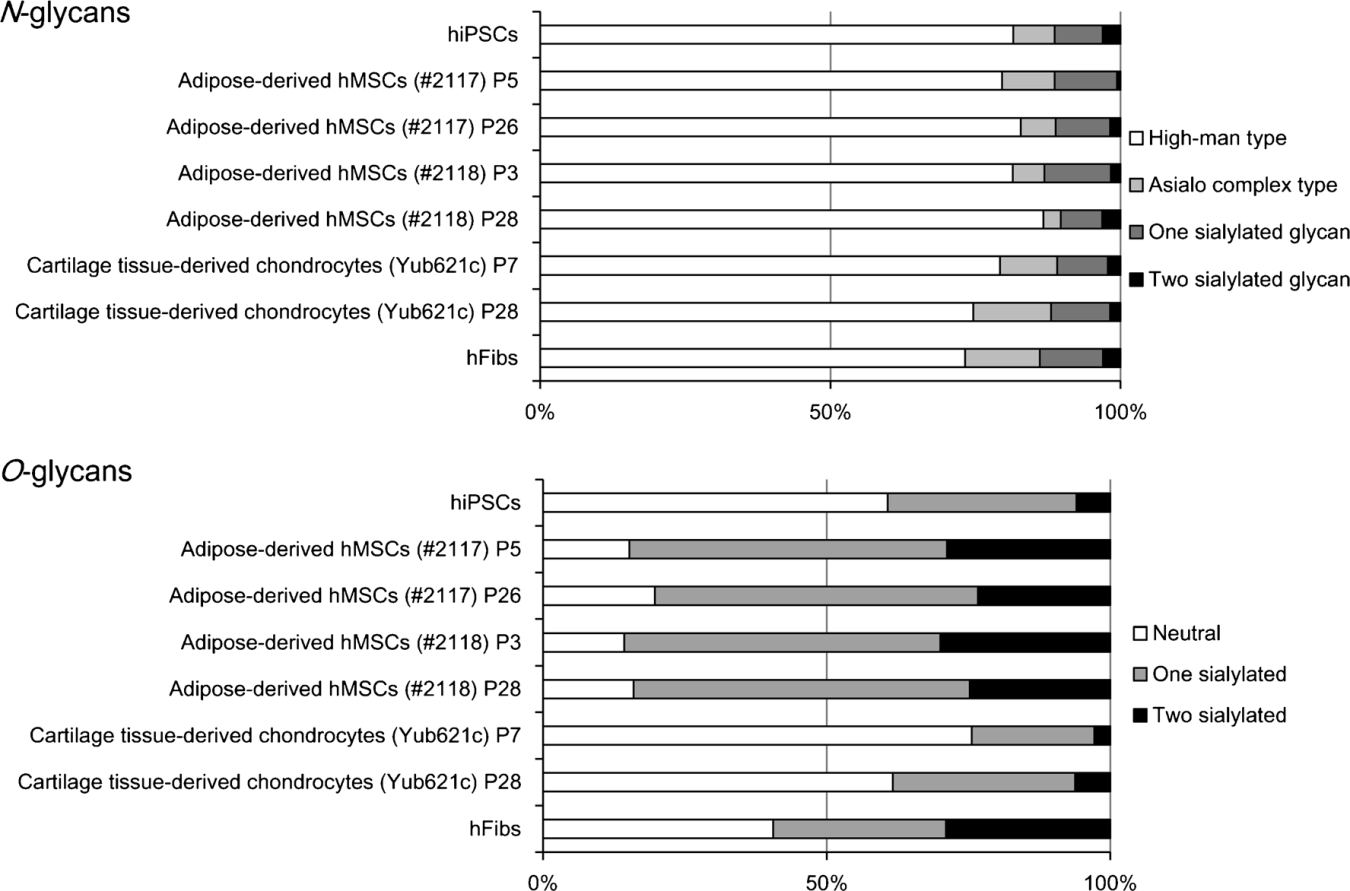
Summary of glycan type. Comparison of the glycan type and proportion in *N*- and *O*-glycans prepared from hiPSCs, adipose-derived hMSCs (lot#: 2117) P5 and P26, adipose-derived hMSCs (lot#: 2118) P3 and P28, cartilage tissue-derived chondrocytes (Yub621c) P7 and P28, and hFibs. Results were calculated based on the data in Tables 1 and 2

Asialo complex type (No.13–16) and sialylated *N*-glycans (No. 17–27) were also detected in significant amounts. A hybrid type *N*-glycan was also observed (No. 23) (Table 1). Among the *O*-glycans, interestingly, a high percentage of sialylated *O*-glycans was detected in adipose-derived hMSCs (Fig. 3). Sialyl Tn (No. 32) gave the highest percentage in both early and late passages of adipose-derived hMSCs (Fig. 2 and Table 2). Disialyl T (Siaα2–3Galβ1–3(Sia*α*2–6)GalNAc, No. 34) was also detected in significant amounts. Core1 (Galβ1–3GalNAc, No. 28) and core2 [Galβ1-4GlcNAcβ1–6(Galβ1–3)GalNAc, No. 29], and their sialylated forms (No. 30,31, 33, 34) were also detected. The percentage of sialyl Tn (Sia*α*2–6GalNAc, No. 32) was observed to be higher in late passage cells (lot#: 2117 P26 and lot#: 2118 P28) compared with early passage cells (lot#: 2117 P5 and lot#: 2118 P3).

### Comparative analysis of *N*- and *O*-glycans of adipose-derived hMSCs with other cell types

Overall signatures of *N*- and *O*-glycans of adipose-derived hMSCs were compared to hiPSCs (cell line 201B7) as a representative of human pluripotent stem cells, and to hFibs as a representative of somatic cells, in addition to another type of somatic stem cell, cartilage tissue-derived chondrocytes (Yub621c, P7 and P28) (Fig. 3). All of the cells gave similar patterns of *N*-glycan types. As described above, high-mannose type *N*-glycans gave the highest percentage among the types of *N*-glycans that were present. Asialo and sialylated *N*-glycans were also observed.

In contrast, the overall *O*-glycan signatures were different depending on the cell types under consideration. Interestingly, adipose-derived hMSCs gave a high percentage of sialylated *O*-glycans (average 84 %) relative to hiPSCs (39 %), hFibs (59 %), and cartilage-derived chondrocytes (31 %).

### α2–6-sialylated *N*-glycans, but not *O*-glycans, are markers of the differentiation potential of stem cells

**Fig. 4:**
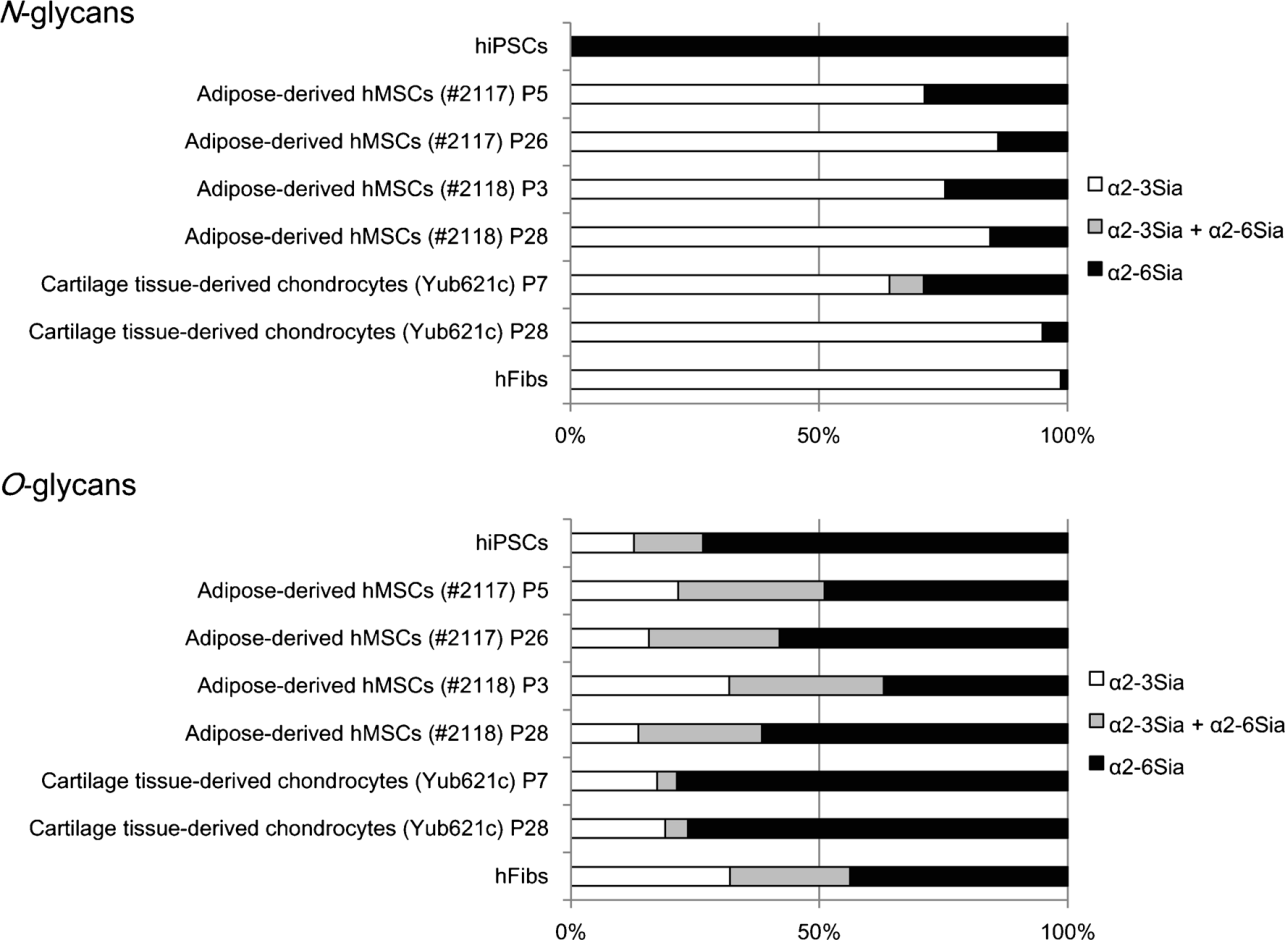
Linkage mode of Sia. Comparison of the Sia linkage type in *N*- and *O*-glycans prepared from hiPSCs, adipose-derived hMSCs (lot#: 2117) P5 and P26, adipose-derived hMSCs (lot#: 2118) P3 and P28, cartilage tissue-derived chondrocytes (Yub621c) P7 and P28, and hFibs. Data are expressed as a percentage of the total amount of sialylated *N*- or *O*-glycans obtained from each sample (taken as 100 %). Results were calculated based on the data provided in Tables 1 and 2

We subsequently focused our attention on sialylation, since α2–6Sia-binding lectins showed stronger binding to early passage hMSCs derived from adipocytes and bone marrow than their corresponding late passage cells, as previously reported [8]. Figure 4 summarizes the Sia linkage type in *N*- and *O*-glycans, where “α2–3Sia” and “α2–6Sia” indicate *N*- or *O*-glycans containing α2–3Sia and α2–6Sia, respectively, and “α2–3Sia + α2–6Sia” indicate *N*- or *O*-glycans containing both α2–3Sia and α2–6Sia.

In terms of *N*-glycans, 201B7 hiPSCs expressed exclusively α2–6Sia (100 %), whereas, in contrast, hFibs expressed mostly α2–3Sia (1 % for α2–6Sia) [17]. In agreement with the previous report [8], the percentage of α2–6-sialylated *N*-glycans (Fig. 4, *black* and *grey*) was indeed higher in early passage adipose-derived hMSCs (29 % for lot#: 2117 P5, 25 % for lot#: 2118 P3) than for corresponding late passage cells (14 % for lot#: 2117 P26, 16 % for lot#: 2118 P28). Similarly, early passage cartilage tissue-derived chondrocytes (29 % for P7) expressed a higher percentage of α2–6-sialylated *N*-glycans than corresponding late passage cells (5 % for P28). A major *α*2–6-sialylated *N*-glycan structure detected in adipose-derived hMSCs and cartilage tissue-derived chondrocytes was “mono-sialylated biantennary *N*-glycan” (Fig. 2 and Table 1). *O*-glycans containing *α*2–6Sia such as sialyl Tn (Sia*α*2–6GalNAc) and disialyl T (Siaα2–3Galβ1–3(Sia*α*2–6)GalNAc) were also detected in *O*-glycans (Table 2). However, no significant relationship was observed between the differentiation potential of stem cells and the Sia linkage mode of *O*-glycans. Taken together, these results clearly demonstrate that *α*2–6-sialylated *N*-glycans, but not *O*-glycans, are markers of the differentiation potential of stem cells.

## Discussion

Previously, we performed a quantitative glycome analysis targeting both *N*- and *O*-glycans derived from 201B7 hiPSCs and hFibs representing undifferentiated and differentiated cells, respectively, using the same strategy described in the present report [17]. A dramatic glycome shift became evident upon conversion from differentiated hFibs to undifferentiated hiPSCs. One of the most significant changes was the Sia linkage mode, which for *N*-glycans of 201B7 hiPSCs was found to consist exclusively of α2–6Sia, whereas that of hFibs was mostly of the α2–3Sia type [17]. Recently, using the systematic glycan profiling system called high-density lectin microarray, we found that α2–6Sia-specific lectins (SNA, SSA, TJA1, and rPSL1a) show stronger binding to early passage cells (with differentiation ability) than late passage cells (without this ability) [8]. Similar results were observed for bone marrow-derived hMSCs and cartilage tissue-derived chondrocytes. Furthermore, the removal of Sia by sialidase treatment significantly reduced the differentiation efficiency of hMSCs. Therefore, we proposed that α2–6-sialylation could be a functional marker of the differentiation potential of stem cells. In the present report, we have performed a structural and quantitative analysis of the glycome of early and late passages of adipose- and cartilage tissue-derived chondrocytes using HPLC analysis combined with MS. We clearly demonstrate that the percentage of α2–6Sia-containing *N*-glycans, but not *O*-glycans, was found to be higher in early passage cells than late passage cells. Therefore, *α*2–6-sialylaed *N*-glycans could serve as markers of the differentiation potential of stem cells.

SNA and SSA, but not TJA1 and rPSL1a, bound to bovine submaxillary mucins expressing sTn as described in the previous report [8]. Therefore, sTn could be target glycans for SNA and SSA, although sTn showed no relationship with the differentiation capacity of hMSC. In this sense, TJA1 and rPSL1a without the binding affinity to sTn might be better probes for the purpose of the evaluation of the differentiation capacity of hMSCs.

The expression of α2–6-sialyltransferase (ST6Gal-I) has been shown to play an important role in the regulation of cellular pluripotency in human pluripotent stem cells [18–20]. Therefore, the key phenomena might be the changes of the expression of ST6Gal-I. ST6Gal-I catalyzes the addition of terminal α2–6Sia to *N*-glycans, but not *O*-glycans. This might be the reason why α2–6–sialylation on *N*-glycans, but not *O*-glycans, changes depending on the differentiation potential of hMSCs. Combined with our findings showing that α2–6Sia is dominant on *N*-glycans of hiPSCs and correlates with the differentiation capacity of cells, it seems that the expression of α2–6-sialylated *N*-glycans is associated with “stemness”.

What are the roles of α2–6Sia in stem cell functions? Previously we showed that α5integrin with 14 *N*-glycosylation sites is one of the carrier proteins of α2–6Sia in cartilage tissue-derived chondrocytes [8]. α5β1integrin is a well-known fibronectin receptor, and its interaction with fibronectin is essential for cell migration and proliferation, for the activation of intracellular signaling pathways, and cytoskeletal formation [21]. Therefore, α2–6Sia on α5integrin could modulate interactions with matrix components such as fibronectin and regulate the differentiation potency of stem cells. Further studies are required to understand the roles of α2–6Sia in this process.

The development of novel markers to evaluate the properties of hMSCs is keenly required for the realization of safe stem cell-based therapy, with such cells being used as hCTPs in regenerative medicine to treat various diseases. Here we have provided structural evidence showing that the expression of α2–6-sialylated *N*-glycans varies depending on the differentiation potential of stem cells. Rapid, sensitive, and nondestructive process analytical testing (PAT) is essential for the stable supply of hMSCs, which could be used for monitoring during the cell production process. The quality control system to assess hMSCs for use as CTPs will become increasingly more important and stringent from the viewpoint of regulatory science.
